# Michael Graham Gelder MA, DM Oxon, FRCP, FRCPsych (Hon), FMedSci, DPM

**DOI:** 10.1192/bjb.2018.36

**Published:** 2018-10

**Authors:** Philip J. Cowen

Formerly Foundation Professor of Psychiatry, University of Oxford, UK


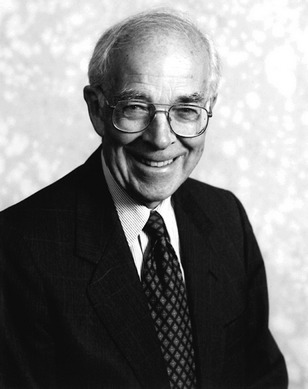


As a young clinical researcher at the Institute of Psychiatry in London in the 1960s, Michael Gelder was only too aware of the need for better treatments of the ‘neuroses’. Management of these conditions was limited to dependence-producing anxiolytic medications and lengthy psychoanalytic psychotherapies, neither of which were particularly effective. Around this time, psychologists had begun to apply learning theory to clinical anxiety disorders, which were conceived as a manifestation of faulty learning or, more specifically, the failure to ‘extinguish’ anxiety that was contextually inappropriate. However, it was not until Gelder's work that these ideas influenced mainstream psychiatry. Gelder, with his characteristic intellect and scientific rigour, developed effective clinical methods to lessen anxiety by ‘desensitisation’, in which gradual exposure to the feared stimulus was coupled with physical relaxation. This resulted in his seminal 1966 publication with Isaac Marks, which described the first controlled trial of this form of therapy in patients with severe agoraphobia.^1^

A few years later, Gelder's growing reputation as someone able to carry out original and clinically important research across disciplinary boundaries led him to be recruited to the Foundation Chair in Psychiatry at the University of Oxford. Arriving at the Warneford Hospital in 1969, Gelder's task was to establish from scratch, and with limited resources, a world-class psychiatric research department, as well as organising and conducting undergraduate and postgraduate teaching and sharing in the clinical work of the hospital.

These goals were formidable, but Oxford had chosen well. Rather unusually for a brilliant researcher and clinician, Gelder also possessed remarkable organisational abilities and committee skills, which he used tirelessly and selflessly to build a thriving Department of Psychiatry, one recognised as a world-leading centre for the development and clinical application of psychological treatments.^2^ Early work on agoraphobia used home-based methods to enhance the effectiveness of the behavioural therapy he had developed at the Institute of Psychiatry. Although this was a major advance, Gelder was quick to realise the limitations of a purely behavioural approach and encouraged exploration of the value of adding cognitive strategies, focusing on modifying thoughts, attention and memory. The Oxford Centre that he led was distinguished by an unusually close interplay among psychological theories, experimental studies and clinical innovation – a particularly productive approach. New and highly effective forms of cognitive–behavioural therapy were developed for panic disorder, generalised anxiety disorder, social anxiety disorder, obsessive–compulsive disorder, hypochondriasis, post-traumatic stress disorder, chronic fatigue syndrome and bulimia nervosa. These cognitive–behavioural treatments have been widely adopted in clinical practice, are recommended by the National Institute for Health and Care Excellence and provide better long-term outcomes than alternative approaches such as antidepressant medication. They have benefited enormous numbers of people worldwide.

In addition to his personal interest in psychological treatments, together with David Grahame-Smith, Nuffield Professor of Clinical Pharmacology, Gelder developed a research unit for the equally new field of psychopharmacology. At the time, this was an almost unique example of cross-departmental collaboration in the Oxford Medical School. The unit's work on the mechanisms by which treatments such as electroconvulsive therapy, anxiolytics and antidepressants actually work has fundamentally shaped our understanding of the biology underlying psychiatric disorders.

Michael Gelder was born in 1929 in Ilkley, the only child of Philip Gelder, a wool merchant, and Alice Gelder, the daughter of a general practitioner. The family soon moved to Bradford, where Gelder attended the local grammar school. Gelder had a longstanding desire to study medicine, but his parents discouraged this ambition, arguing that he should join his father in the family business. However, Gelder persisted, taking science A-levels at evening classes and then winning a scholarship to Queen's College, Oxford, where he took first class honours in physiology. A further scholarship to University College Hospital allowed him to complete his medical training, which was followed by National Service as a Medical Officer at the British Army of the Rhine Headquarters from 1956 to 1958. Through his medical training and early clinical work, Gelder became intrigued by the pervasive role of psychological factors in medical practice. This led him to embark on his training in psychiatry at the Maudsley Hospital, during which he won the prestigious Gaskell Gold Medal of the Royal College of Psychiatrists. Strongly encouraged by Professor Aubrey Lewis, he then worked as a Medical Research Council Research Fellow at the Institute of Psychiatry.

Gelder led the Oxford Department of Psychiatry until his retirement in 1996. He was notable for ‘leading from the front’, attending all departmental academic meetings, as well as carrying a significant clinical and teaching load. His unparalleled lucidity of thought and encyclopaedic knowledge made him an inspirational teacher; this was captured particularly in the Oxford Textbook of Psychiatry, which he co-authored with Dennis Gath and Richard Mayou. Translated into six languages, this became the standard textbook for psychiatric trainees. One of his most important priorities was to foster the talented young clinicians and scientists who joined his department. Many went on to be leaders in the psychiatric and research communities.

Although Gelder served on major research committees of the Medical Research Council and Wellcome Trust, he had no interest in the personal accumulation of power, and was self-deprecating and modest. Invariably dressed in a grey suit and tie – even when walking his Alsatian dogs – he had a formality of manner, which, coupled with his intense drive for clinical and scientific excellence and his formidable intellect, could make him a somewhat forbidding figure. However, patients spoke of his approachability and kindness, while trainees would comment on how his acute clinical perception and genuine concern for them helped them cope with and learn from the most difficult of situations.

This side of Gelder's nature was, of course, well-known to his family. His daughter Fiona remarked that, as a father, ‘he was kind, fair and unfailingly supportive of us all – in school life, marriage and work decisions’. She is a general practitioner, Colin is a chest physician and Nicola runs her own business. Gelder met his wife, Mandy, when he was a medical student at University College Hospital and she was a nurse. Gelder regarded persuading her to marry him as his greatest achievement, and he was devoted to her throughout their nearly 64 years of marriage, showering her with flowers on anniversaries and Valentine's Days.

In retirement, Gelder was able to find more time to indulge his love of travel. He had a wide circle of fond and loyal friends who would have been astonished to learn of the trepidation he had sometimes engendered in junior colleagues. When he gained an Italian son-in-law, he joined Italian classes and at the wedding gave a speech in both English and fluent Italian. Until arthritis supervened, he continued to play real tennis at Merton College, where he was a Fellow. He was an affectionate and attentive grandfather to his eight grandchildren, one of whom, to his delight, recently qualified in medicine.
